# Introduced Populations of an Invasive Tree Have Higher Soluble Sugars but Lower Starch and Cellulose

**DOI:** 10.3389/fpls.2020.587414

**Published:** 2020-10-15

**Authors:** Wenrao Li, Luwei Wang, Baoliang Tian, Jianqing Ding, Evan Siemann

**Affiliations:** ^1^State Key Laboratory of Crop Stress Adaptation and Improvement, School of Life Sciences, Henan University, Kaifeng, China; ^2^Department of Biosciences, Rice University, Houston, TX, United States

**Keywords:** Chinese tallow tree, soluble sugars, starch, cellulose, root water potential

## Abstract

Native and introduced plant populations vary in leaf physiology, biochemistry, and biotic interactions. These aboveground traits may help invasive plants in competition for resources with co-occurring native species. Root physiological traits may affect invasive plant performance because of the roles of roots in resource absorption. The aim of this study was to test this prediction, using invasive Chinese tallow tree (*Triadica sebifera*), as a model species. Here we examined carbohydrate (soluble sugar, sucrose, fructose, starch, and cellulose) concentrations and the mass of roots, stems, and leaves, along with root water potential and arbuscular mycorrhizal fungi (AMF) colonization of soil-cultured *T. sebifera* seedlings from 10 native (China) and 10 introduced (United States) populations in a common garden. Introduced populations had a significantly greater stem and leaf mass than native populations but their root masses did not differ, so they had lower R:S. Introduced populations had higher soluble sugar concentrations but lower starch and cellulose concentrations in their leaves, stems, and roots. Introduced populations had more negative root water potentials and higher AMF colonization. Together, our results indicate that invasive plants shift their carbohydrate allocation, leading to faster growth and a greater aboveground allocation strategy. Higher AMF colonization and more negative water potential in invasive plants likely facilitate more efficient water absorption by the roots. Thus, such physiological variation in root characteristics could play a role in plant invasion success.

## Introduction

Successful invasive plants often optimize their performance and competitive ability by improving resource acquisition and use in their introduced ranges (Zou et al., 2007; Feng et al., 2009; Chen et al., 2013; Liu et al., 2017; Petruzzellis et al., 2019). However, nearly all studies of variation among native and introduced populations of invasive plants focus on foliar ecophysiology, while knowledge of the roles of root ecophysiological traits in facilitating biological invasion is still limited. Roots play a vital role in determining plant growth, development, and adaptation via water uptake, nutrient acquisition and carbon storage and exudation (McCormack et al., 2015; Erktan et al., 2018; Lü et al., 2018; Petruzzellis et al., 2019). Whether plants from introduced populations have different root physiological traits compared with congeneric native populations, and whether these differences contribute to the faster growth of invasive plants are largely unexplored.

Furthermore, invasive plant root growth and functioning are affected by the synthesis, allocation, and storage of carbohydrates. Carbohydrates in plant tissues are usually classified as structural or non-structural carbohydrates according to their chemical structure and function in plants (Dietze et al., 2014). Structural carbohydrates, as non-storage components, are long-chained molecules and mainly include polymerized compounds, such as cellulose which confers mechanical strength to cells, playing important roles in maintaining or changing plant tissue density (Dumas et al., 2008; Miedes et al., 2014). Thus, low cellulose concentrations in roots may reduce defense against natural enemies, but facilitate arbuscular mycorrhizal fungi (AMF) penetrating into roots, increasing AMF colonization. AMF establish symbioses with most terrestrial plants, assisting plants to obtain water and nutrients from the soil by forming a large composite network of root hyphae to expand the absorption area of the host plant roots and increase the root length, and by inducing greater aquaporin expression (Simard et al., 2012; Ruiz-Lozano and Aroca, 2017; Lü et al., 2018). Studies have shown some introduced populations of plants can have higher AMF colonization than native ones (Nijjer et al., 2008; Yang et al., 2015a,b). However, it remains unknown whether cellulose concentrations differ between introduced and native populations despite the potential for this difference to play a role in invasion success.

Non-structural carbohydrates include soluble sugars (such as glucose, sucrose, or fructose) and starch, which serve different physiological functions. Starch is a long-term carbon storage pool because of its osmotic inactivity (Hartmann and Trumbore, 2016), while soluble sugars are used directly for cellular metabolism, as the substrates of cellular respiration or osmolytes for maintaining plant growth (Rosa et al., 2009), and provide the energy for AMF colonization (Lü et al., 2018; Rodríguez-Caballero et al., 2018). Non-structural carbohydrates can undergo frequent transformations including the conversion of soluble sugar to starch for storage or the conversion of starch to soluble sugar for metabolism. Therefore, tissue soluble sugar and starch maintain a dynamic trade-off between growth (defense, osmoregulation, and symbiosis) and storage (Dietze et al., 2014; Hartmann and Trumbore, 2016), which represent different adaptation strategies to various environments. Hinman and Fridley (2018) found that native species have higher root soluble sugar concentrations and employ a grow-first strategy prioritizing allocation to new structural biomass over carbon storage, but invaders tended to store more root starch for new root growth in spring. A cross-continental comparison of sycamore maple (*Acer pseudoplatanus*) showed trees from native populations had higher foliar glucose and fructose concentrations than invasive population trees but their sucrose and starch concentrations were similar (Shouman et al., 2017). To date, however, there are few reports examining the root structural and non-structural carbohydrate traits of introduced and native populations of invasive plants (Lin et al., 2018). Testing such differences may reveal whether invasive plants shift their root physiological strategies after they are introduced from their home range.

Here we examined the mass and carbohydrate concentrations of roots, stems, and leaves as well as root water potential and AMF colonization in a common garden experiment with plants of Chinese tallow trees (*Triadica sebifera*) from China (native range) and USA (introduced range). Specifically, we asked the following questions: (1) How do soluble sugars, starch, and cellulose concentrations vary among leaves, stems, and roots of introduced and native populations? (2) How do their AMF colonization and root water potential differ? We predict that, relative to native populations, introduced populations have a greater allocation to soluble sugars vs. starch and cellulose, higher AMF colonization and more negative root water potentials.

## Materials and Methods

### Experimental Set-Up

Chinese tallow tree (*Triadica sebifera*) is native to China and was introduced to the United States in the late 18th century where it has become invasive throughout the southeast (Pile et al., 2017). Relative to native conspecifics, introduced populations of *T. sebifera* have more rapid growth, larger leaf area or specific leaf area, greater photosynthetic rates (Zou et al., 2006, 2007; Chen et al., 2013; Yang et al., 2015b), higher rates of AMF association (Yang et al., 2015a,b, Pei et al., 2020), and greater plasticity of functional and fitness-related traits under salt stress (Chen et al., 2013; Zhang et al., 2013; Yang et al., 2015a). Previous studies have also reported that plants of Chinese tallow tree had higher AMF colonization than several native plant species in the introduced areas (Nijjer et al., 2008; Paudel et al., 2014).

In the fall of 2016, we hand collected seeds from trees in 10 populations across the native range and 10 populations across the introduced range (Supplementary Table S1). We collected mature seeds (pods had dehisced to expose seeds) from 6 to 10 widely spaced, haphazardly selected adult trees. We pooled seeds from separate trees within each population. The introduced populations included those descended from the two historically recorded major introduction events (Pile et al., 2017) along with likely source populations as identified in a microsatellite study (DeWalt et al., 2011). The Georgia populations are descended from the original introduction in 1772, which was most likely from a Guangdong province population and the rest of the US populations are descended from a later introduction around 1900 most likely from a Jiangsu province population (DeWalt et al., 2011).

We removed the seeds’ waxy coats (by soaking them in water with detergent then scrubbing), surface sterilized them (HgCl_2_ [0.1%] for 1 min) and rinsed them repeatedly with distilled water. Then we put them in moist sand at 4°C for ∼30 days. Following this, we sowed ∼300 seeds of each population in a tray filled with a mixture of vermiculite and commercial topsoil that we placed in an unheated greenhouse.

We conducted our experiments in Kaifeng, Henan, China. Due to logistical constraints related to measuring root water potentials, we split the experiment into two-time blocks. We filled PVC tubes (24.5 cm high, diameter 6.3 cm; 32 tubes in July and 64 tubes in August 2017) with 630 g of soil and vermiculite (volume 2:1). We collected soil from a field left fallow for three years. We separately collected soil for the July (1.0 g kg^–1^ total nitrogen, 9.2 mg kg^–1^ available phosphorous, 178.8 mg kg^–1^ available potassium, 5.0 g kg^–1^ organic carbon) and August (1.4 g kg^–1^ total nitrogen, 14.7 mg kg^–1^ available phosphorous, 173.7 mg kg^–1^ available potassium, 10.6 g kg^–1^ organic carbon) blocks to avoid storing soil.

We planted one seedling (three-leaf stage) into each PVC tube. At each time block we transplanted four seedlings for a population (eight for GL) for mass, carbohydrate and root water potential measurements and another set of four seedlings (eight for GL) in August only for AMF measurements. We randomly arranged tubes in a mesh-sided greenhouse, watered them daily, and changed their positions every other day. Light and temperature approximated ambient conditions. At the beginning of the experiment, all seedlings used were similar in size. We allowed seedlings to grow from July 12 to August 24, 2017 or from August 12 to October 4, 2017.

### Root Water Potential

We placed each tube individually into a pressure chamber (Model: 3005, Soil moisture Equipment Corp., CA, United States), then we removed their leaves and clipped their stems slightly (∼2.5 cm) above ground level. We sealed the pressure chamber lid around the stem and raised the pressure in the chamber until tiny droplets appeared on the cut surface. The pressure point indicates root water potential.

### Mass

After measuring root water potential, we clipped the remaining stem at ground level. Then we washed roots out of the soil. We dried the leaves, stems and roots and weighed them. The dry weight of every organ was defined as leaf, stem, and root mass, respectively.

### Carbohydrate Content

We separately ground each plant’s roots, stem, and leaves (excluding petiole) using a ball mill (Retsch-MM400, Retsch, Haan, Germany). The tissue total soluble sugar, sucrose, and fructose concentrations as well as starch and cellulose concentrations were determined using Comin Biochemical Test Kits (Comin Biotechnology Co., Ltd., Suzhou, China) per the manufacturer’s instructions. For a complete measurement of soluble sugar and starch concentrations, the sample was extracted three times and then the supernatant was used for soluble sugar determination and the residue was used for starch measurement.

### AMF

We examined AMF on the fine root samples from the other set of plants. We stored each AMF root sample in a histocassette in ethanol until we processed them following the methods of Nijjer et al. (2004, 2008). We cleared and bleached the roots. Then we acidified and stained them (trypan blue), and slide-mounted 30 1-cm fragment of fine roots (PVA mount). We examined the slides (400× magnification) and recorded the number of 300 grid intersection points at which mycorrhizal hyphae were present.

### Statistical Analyses

We used a series of ANOVAs (PROC GLM, SAS 9.4) with time block, population origin, population nested in origin and their interactions as fixed factors to examine the variation in mass and carbohydrates of roots, stems and leaves as well as root water potential. We used the variation among populations to test for a significant origin effect and we used the interaction of populations and time block to test for a significant origin by block effect. We conducted an additional ANOVA without any time block terms to examine AMF colonization (only measured in August block).

## Results

### Mass

**FIGURE 1:**
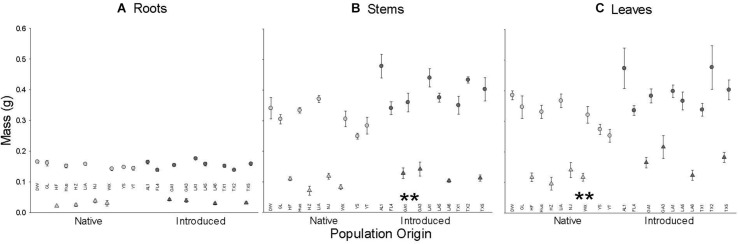
The dependence of root **(A)**, stem **(B)**, and leaf **(C)** mass on population origin (light fill is native, dark fill is introduced). Each point is the means ± SE of a population (abbreviations from Supplementary Table S1) in the July (triangle) or August (circle) time block. ** indicates *P* < 0.01 for the main effect of population origin (see Table 1 for full results). *N* = 4 except for population GL (*N* = 8).

Plants from introduced populations had a significantly greater stem and leaf mass than those from native populations but their root masses did not differ (Figure 1 and Table 1); thus, they had lower root to shoot ratio (R:S) (introduced: 0.162 ± 0.007; native 0.192 ± 0.007; *F*_1_,_18_ = 5.2, *P* = 0.0344). Similarly, leaf and stem masses varied with population(origin), but root mass did not (Table 1 and Figure 1). From visual inspection, there were no apparent effects of introduction event of an introduced population on any mass variables (GA1 and GA3 vs. rest of introduced populations, Figure 1). Plants were larger in the August block (Figure 1) but there were no significant interactions between the origin and time block or population(origin) and time block (Table 1). However, the incomplete, unbalanced design to populations (Supplementary Table S1) provided little power to examine the interaction of origin or population(origin) with time block.

**TABLE 1:** The dependence of mass, physiological, and chemical properties of *T. sebifera* plants (roots, stems, and leaves) on population origin, population nested in origin, experimental block and their interactions as fixed effects.

	**Origin**		**Pop(origin)**		**Block**		**Origin × block**		**Pop × block(origin)**	
**Variable**	**F_1_,_18_**	**P**	**F_1_,_73_**	**P**	**F_1_,_73_**	**P**	**F_1_,_1_**	**P**	**F_1_,_73_**	**P**
Roots										
Mass	0.7	0.4203	2.6	0.0021	**497.6**	**< 0.0001**	0.3	0.6639	1.4	0.2431
Total soluble solids	**52.5**	**< 0.0001**	1.1	0.4181	**14.7**	**0.0003**	< 0.1	0.8996	0.7	0.4072
Fructose	**15.2**	**0.0011**	0.8	0.6512	2.5	0.1214	3.5	0.3134	1.3	0.2517
Sucrose	**14.0**	**0.0015**	**1.9**	**0.0321**	**16.2**	**< 0.0001**	0.9	0.5128	0.3	0.6030
Starch	**15.6**	**0.0009**	**4.0**	**< 0.0001**	**7.7**	**0.0072**	67.4	0.0772	0.1	0.7104
Cellulose	**10.2**	**0.0050**	**1.8**	**0.0491**	**7.9**	**0.0062**	0.2	0.7629	0.4	0.5304
Water potential	**7.3**	**0.0146**	0.6	0.8965	**37.9**	**< 0.0001**	0.1	0.8416	5.5	0.0218
Stem										
Mass	**11.9**	**0.0029**	**3.2**	**0.0002**	**177.7**	**< 0.0001**	0.5	0.5965	1.8	0.1853
Total soluble solids	**18.3**	**0.0005**	1.5	0.1130	**10.0**	**0.0022**	< 0.1	0.8829	**7.3**	**0.0086**
Fructose	**4.7**	**0.0438**	0.7	0.8152	0.5	0.4782	0.8	0.5393	**6.0**	**0.0168**
Sucrose	**11.7**	**0.0030**	1.5	0.1173	**31.1**	**< 0.0001**	1.7	0.4154	0.2	0.6464
Starch	3.8	0.0660	2.0	0.0176	**77.9**	**< 0.0001**	31.9	0.1115	< 0.1	0.9652
Cellulose	**21.8**	**0.0002**	0.9	0.6293	2.9	0.0927	42.8	0.0965	0.1	0.8081
Leaves										
Mass	**9.6**	**0.0061**	**2.1**	**0.0135**	**61.8**	**< 0.0001**	55.8	0.0847	< 0.1	0.9710
Total soluble solids	**31.4**	**< 0.0001**	**1.9**	**0.0317**	0.2	0.6332	6.3	0.2412	0.5	0.5000
Fructose	**47.2**	**< 0.0001**	0.5	0.9439	1.7	0.1939	0.2	0.7532	2.1	0.1499
Sucrose	**13.5**	**0.0018**	**1.9**	**0.0285**	2.5	0.1206	5.7	0.2529	0.4	0.5488
Starch	**12.3**	**0.0025**	1.5	0.1183	**7.7**	**0.0072**	0.4	0.6348	1.1	0.3081
Cellulose	**12.7**	**0.0022**	**3.1**	**0.0003**	**7.7**	**0.0072**	**782.7**	**0.0227**	< 0.1	0.9117

*The imbalanced design (Supplementary Table S1) resulted in little power to test for effects of Origin × block or Pop × block(origin). Significant results indicated in bold.*

### Carbohydrate Content

**FIGURE 2:**
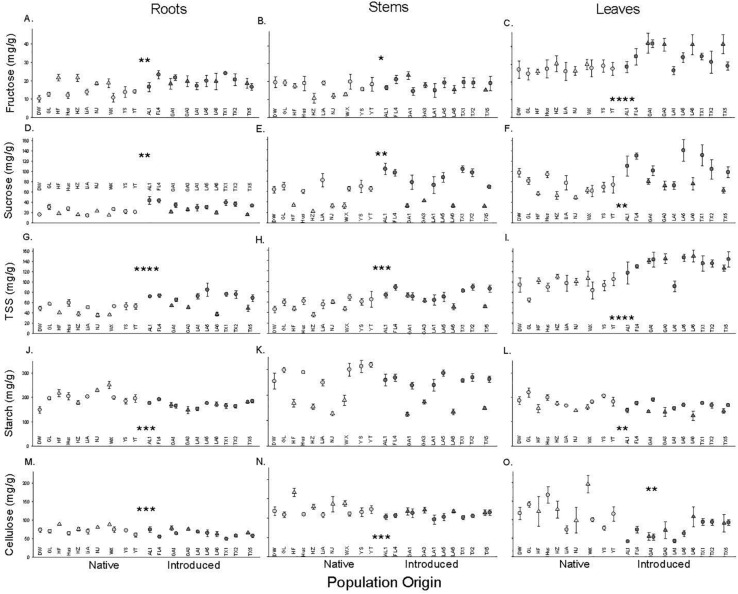
The dependence of root **(A,D,G,J,M)**, stem **(B,E,H,K,N)**, and leaf **(C,F,I,L,O)** carbohydrates (Fructose, **A–C**; Sucrose, **D–F**; Total soluble sugar, **G–I**; Starch, **J–L**; Cellulose, **M–O**) on population origin (light fill is native, dark fill is introduced). Each point is the mean ± SE of a population (abbreviations from Supplementary Table S1) in the July (triangle) or August (circle) time block. * indicates *P* < 0.05, ** indicates *P* < 0.01, *** indicates *P* < 0.001, **** indicates *P* < 0.0001 for the main effect of population origin (see Table 1 for full results. *N* = 4 except for population GL (*N* = 8).

Introduced populations had higher concentrations of total soluble sugars, fructose [except stems], and sucrose but lower concentrations of starch and cellulose in their leaves, stems and roots than native populations (Figure 2 and Table 1). Populations varied in some of these characteristics with an especially significant variation for root starch and leaf cellulose (Figure 2 and Table 1). Some of the variables depended on the time block. In August, sugars were significantly higher, but cellulose was lower in roots and stems, while leaf sugar and starch concentrations were higher and cellulose was lower. Only leaf cellulose depended on origin × time block with a larger difference between native and introduced populations in July than August (+ 55 vs. + 47 mg/g, Figure 2).

### Root Water Potential and AMF Colonization

**FIGURE 3:**
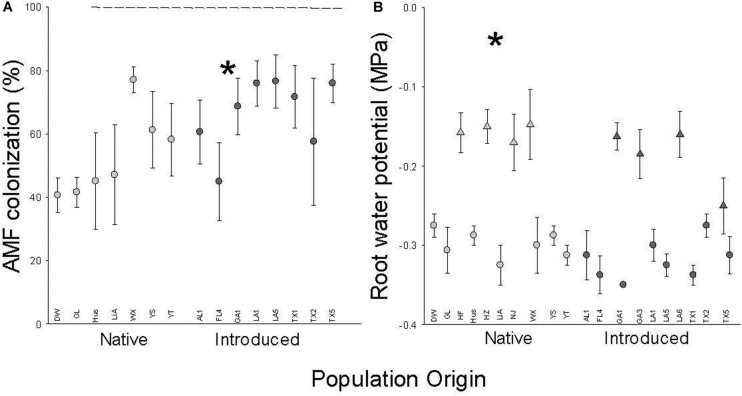
The dependence of AMF colonization **(A)** and root water potential **(B)** on population origin (light fill is native, dark fill is introduced). Each point is the mean ± SE of a population (abbreviations from Supplementary Table S1) in the July (triangle) or August (circle) time block. * indicates *P* < 0.05 for the main effect of population origin (see Table 1 for full results. *N* = 4 except for population GL (*N* = 8).

Introduced populations had more negative root water potentials and higher rates of AMF colonization (*F*_1_,_13_ = 4.8, *P* = 0.0483) than native populations (Figure 3 and Table 1). Both root water potential and AMF colonization did not significantly vary with population (origin) (AMF: *F*_13_,_49_ = 1.4, *P* = 0.2085; Figure 3 and Table 1). Root water potentials were less negative in the July block (Figure 3 and Table 1) but there were no significant interactions between population origin and time block or population(origin) and time block.

## Discussion

Several studies have compared leaf morphological, physiological, and biochemical traits of plants from native and introduced populations (Feng et al., 2009; Angelo and Pau, 2017; Liu et al., 2017; Petruzzellis et al., 2019) but root physiological traits are largely neglected. Root carbohydrate allocation is closely related to plant growth, resource storage, and defense. Here, we found that *T. sebifera* roots of plants from native and introduced populations varied in water potentials, AMF colonization and carbohydrate contents. These findings indicate the differences in those root traits in the introduced range may play a role in the more rapid shoot growth of plants from introduced populations, which helps to better understand the invasion success of this species and likely other plant species.

In this study, the greater concentrations of soluble sugars together with lower concentrations of starch and cellulose in *T. sebifera* plants from introduced populations suggest a shift from storage and defense to more rapid metabolism in the introduced range. Our results also support a trade-off between the allocation to soluble sugars and starch, given starch and sugar are often in a dynamic conversion state. Soluble sugars are strongly positively associated with more active tissue metabolism and rapid growth (Dietze et al., 2014), while starch is the most common storage carbohydrate (Hartmann and Trumbore, 2016; Thalmann and Santelia, 2017). Therefore, when the plants had a strong demand to grow, less starch will be synthesized for decreasing energy store, and more sugar will be synthesized for increase metabolic energy. In part, these changes reflect that, for *T. sebifera* plants from introduced populations in this study, more soluble sugar might be associated with the decreasing tissue water potential (i.e., more negative) (Tardieu et al., 2018). Similarly, cellulose is a macromolecular polysaccharide composed of glucose such that higher cellulose concentrations limit allocation to other types of carbohydrates. The lower cellulose for lower tissue density perhaps also reflects less need for physical defense or lower risk of tissue loss with low enemy abundance in the introduced range (Hartley et al., 2010; Fan et al., 2013; Petruzzellis et al., 2019). Therefore, the patterns we found for carbohydrates could reflect selection for increased soluble sugars for more active metabolism (such as to allow more rapid growth) by decreasing starch for storage at least during the seedling stage in our study.

Although there were no significant interactions between time block and population origin, the strong effect of time block on water potential motivates the additional study of possible phenological differences between introduced and native populations in attributes such as growth vs. storage. Phenological variation in carbohydrates may also be critical for linking our results to plant strategies. Other factors could simultaneously affect multiple carbohydrates such as increased soluble sugars to provide nutrients to AMF (Lü et al., 2018; Rodríguez-Caballero et al., 2018) and lower cellulose to decrease root surface resistance to AMF infection. Of course, AMF can help plants enhance water absorption by promoting the expression of aquaporins (Ruiz-Lozano and Aroca, 2017). The greater surface area of AMF hyphae than that of plant roots may also increase the absorption of water and nutrients. Higher AMF colonization might play important role for *T. sebifera* invading into the new areas (Nijjer et al., 2008; Paudel et al., 2014). Higher AMF colonization in introduced *T. sebifera* compared to native populations also had been reported by Yang et al. (2015a). However, it is more difficult for them to cause these same changes in carbohydrates that we found for roots to also occur in leaves and stems.

The patterns of soluble sugars, starch, and cellulose in roots, stems, and leaves in this study indicate that there may be a general functional advantage of a higher metabolic rate, lower storage, and/or physical defense in the introduced range for *T. sebifera*. We found similar root mass and higher leaf and stem mass (and so lower R:S) in introduced populations than in native ones (Figure 1). This indicated that relatively more photosynthetic products were allocated to the shoots for the introduced populations and relatively more mass was allocated to root growth for the native populations. A lower R: S may provide an advantage in the competition for aboveground resources (Reynolds and Pacala, 1993) which shows a greater importance for aboveground resource acquisition and competition in the introduced range that has been found for other species (Feng et al., 2009; van Kleunen et al., 2010; Liu et al., 2017; Petruzzellis et al., 2019). The more negative root water potential of plants from introduced populations may reflect their higher solute (soluble sugars) concentrations (Figure 2) that improve the ability of root system to absorb water from the soil because of soluble sugar’s osmotic regulation role (Woodruff and Frederick, 2011; Li et al., 2018). Moreover, *T. sebifera* trees from introduced populations are more able to tolerate conditions of osmotic stress such as salinity (Chen et al., 2013; Yang et al., 2015b).

Overall, our results support a scenario in which *T. sebifera* in its introduced range has shifted to a strategy of faster growth, greater aboveground allocation, and more efficient root water absorption potential in part from more positive AMF interactions and more negative water potential. Significantly higher seedling biomass in introduced *T. sebifera* plants suggests that AMF more benefit introduced populations of *T. sebifera* relative to its native populations. The changes in carbohydrates from lower starch and cellulose to higher soluble sugars are consistent with greater resource investment to the growth rate in the introduced range. Because several invasive plant species have such a pattern of more rapid growth in introduced populations (van Kleunen et al., 2010; Liu et al., 2017; Petruzzellis et al., 2019), such changes in carbohydrates may also have occurred in those invasive plants. This extends our knowledge of physiological variation in root characteristics between ranges for an invasive plant species, though further studies on changes in the belowground structure and function are needed to fully unveil mechanisms involved in the genetic variation of root ecophysiology in invasive plants.

## Data Availability Statement

All datasets presented in this study are included in the article/Supplementary Material.

## Author Contributions

WL and JD planned and designed the research. WL and LW performed the experiments and conducted the field work. ES analyzed the data. WL, JD, and ES wrote the manuscript. BT and ES collected the seeds. All authors have read and approved the submitted manuscript.

## Conflict of Interest

The authors declare that the research was conducted in the absence of any commercial or financial relationships that could be construed as a potential conflict of interest. The reviewer JZ declared a past co-authorship with one of the authors, ES, to the handling Editor.
