# Supplementary material for: Introduced Populations of an Invasive Tree Have Higher Soluble Sugars but Lower Starch and Cellulose
Source: Front Plant Sci. 2020 Oct 15;11:587414. doi: 10.3389/fpls.2020.587414 (PMC7593253; doi:10.3389/fpls.2020.587414)
Supplement: Supplementary file 1 [file Table_1.DOC]

**SUPPLEMENTAL MATERIAL**

**TABLE S1** – Populations used in this experiment in the July and/or August blocks (numbers indicate the number of replicates). The GA1 and GA3 populations are descended from the original introduction in 1772 which was most likely from a Guangdong province population and the rest of the US populations are descended from a later introduction around 1900 most likely from a Jiangsu province population based on a microsatellite study (DeWalt et al., 2011).

|  |  |  |  |  |  |
| --- | --- | --- | --- | --- | --- |
| **Code** | **Collection location** | **Latitude** | **Longitude** | **July** | **August** |
| China populations | | | | | |
| HF | Hefei (Anhui) | 31°50′ N | 117°09′ E | 4 |  |
| Hus | Huangshan (Anhui) | 30°00' N | 117°59′ E |  | 4 |
| YS | Yangshan (Guangdong) | 24°35′ N | 112°41′ E |  | 4 |
| GL | Guilin (Guangxi) | 25°04′ N | 110°18′ E |  | 8 |
| DW | Dawu (Hubei) | 31°35′ N | 114°14′ E |  | 4 |
| NJ | Nanjing (Jiangsu) | 24°42′ N | 117°03′ E | 4 |  |
| WX | Wuxi (Jiangsu) | 31°36′ N | 120°14′ E | 4 | 4 |
| YT | Yingtan (Jiangxi) | 28°19′ N | 117°03′ E |  | 4 |
| HZ | Hangzhou (Zhejiang) | 30°16' N | 120°08' E | 4 |  |
| LiA | Linan (Zhejiang) | 30°47' N | 120°03′ E |  | 4 |
|  |  |  |  |  |  |
| US populations | | | | | |
| AL1 | Tillmans Corner (Alabama) | 30°35′ N | 88°09′ W |  | 4 |
| FL4 | Callahan (Florida) | 30°59′ N | 81°48′ W |  | 4 |
| GA1 | Hutchinson Island (Georgia) | 32°06′ N | 81°06′ W | 4 | 4 |
| GA3 | St. Simons Island (Georgia) | 31°15′ N | 81°37′ W | 4 |  |
| LA1 | Lake Charles (Louisiana) | 30°14′ N | 93°09′ W |  | 4 |
| LA5 | Pumpkin Center (Louisiana) | 30°28′ N | 90°32′ W |  | 4 |
| LA6 | St. Gabriel (Louisiana) | 30°17′ N | 91°05′ W | 4 |  |
| TX1 | Houston (Texas) | 29°47′ N | 95°02′ W |  | 4 |
| TX2 | La Marque (Texas) | 29°22′ N | 95°02′ W |  | 4 |
| TX5 | Port Arthur (Texas) | 29°53′ N | 94°02′ W | 4 | 4 |
|  |  |  |  |  |  |
